# Replacing saturated fatty acids with polyunsaturated fatty acids increases the abundance of Lachnospiraceae and is associated with reduced total cholesterol levels—a randomized controlled trial in healthy individuals

**DOI:** 10.1186/s12944-022-01702-1

**Published:** 2022-09-26

**Authors:** Vibeke H. Telle-Hansen, Line Gaundal, Nasser Bastani, Ida Rud, Marte G. Byfuglien, Terje Gjøvaag, Kjetil Retterstøl, Kirsten B. Holven, Stine M. Ulven, Mari C. W. Myhrstad

**Affiliations:** 1 Faculty of Health Sciences, Oslo Metropolitan University, St. Olavsplass, Postbox 4, 0130 Oslo, Norway; 2 Department of Nutrition, Institute of Basic Medical Sciences, Faculty of Medicine, University of Oslo, Blindern, P.O. Box 1046, 0317 Oslo, Norway; 3 Nofima -Norwegian Institute of Food, Fisheries and Aquaculture Research, Osloveien 1, 1433 Ås, Norway; 4 Mills AS, Sofienberggt. 19, 0558 Oslo, Norway; 5 The Lipid Clinic, Department of Endocrinology, Morbid Obesity and Preventive Medicine, Oslo University Hospital, Nydalen, P.O. Box 4950, 0424 Oslo, Norway; 6 The Norwegian National Advisory Unit On Familial Hypercholesterolemia, Department of Endocrinology, Morbid Obesity and Preventive Medicine, Oslo University Hospital, Oslo, Norway

**Keywords:** Dietary fat, Polyunsaturated fatty acids, Total cholesterol, Gut microbiota, Lachnospiraceae, Randomized controlled trial

## Abstract

**Background:**

Improving dietary fat quality strongly affects serum cholesterol levels and hence the risk of cardiovascular diseases (CVDs). Recent studies have identified dietary fat as a potential modulator of the gut microbiota, a central regulator of host metabolism including lipid metabolism. We have previously shown a significant reduction in total cholesterol levels after replacing saturated fatty acids (SFAs) with polyunsaturated fatty acids (PUFAs). The aim of the present study was to investigate the effect of dietary fat quality on gut microbiota, short-chain fatty acids (SCFAs), and bile acids in healthy individuals. In addition, to investigate how changes in gut microbiota correlate with blood lipids, bile acids, and fatty acids.

**Methods:**

Seventeen participants completed a randomized, controlled dietary crossover study. The participants received products with SFAs (control) or PUFAs in random order for three days. Fecal samples for gut microbiota analyses and fasting blood samples (lipids, fatty acids, and bile acids) were measured before and after the three-day intervention.

**Results:**

Of a panel of 40 bacteria, *Lachnospiraceae* and *Bifidobacterium* spp. were significantly increased after intervention with PUFAs compared with SFAs. Interestingly, changes in *Lachnospiraceae,* as well as *Phascolarlactobacterium* sp. and *Eubacterium hallii,* was also found to be negatively correlated with changes in total cholesterol levels after replacing the intake of SFAs with PUFAs for three days. No significant differences in SCFAs or bile acids were found after the intervention.

**Conclusion:**

Replacing SFAs with PUFAs increased the abundance of the gut microbiota family of *Lachnospiraceae* and *Bifidobacterium* spp. Furthermore, the reduction in total cholesterol after improving dietary fat quality correlated with changes in the gut microbiota family *Lachnospiraceae*. Future studies are needed to reveal whether *Lachnospiraceae* may be targeted to reduce total cholesterol levels.

**Trial registration:**

The study was registered at Clinical Trials (https://clinicaltrials.gov/, registration identification number: NCT03658681).

## Background

Improving dietary fat quality by exchanging the intake of saturated fatty acids (SFAs) with polyunsaturated fatty acids (PUFAs) has been shown to reduce serum cholesterol levels [1–4] and thereby the risk of cardiovascular diseases (CVDs) [3, 5]. Emerging evidence suggests that gut microbiota play a significant role in human metabolic regulation [6]. The gut microbiota and its host are in a symbiotic relationship by a joint utilization of consumed nutrients, and the human gut microbiota with its fermentation products are hypothesized to play a major role in host energy and substrate metabolism [7–9]. Hence, dietary habits affect both the abundance and composition of gut microbiota and host health [10–12]. Diet is one of the most important factors affecting the gut microbiota, and changes in diet can alter the gut microbiota within one to three days [13–16]. Recent studies have suggested that dietary fat is a potential modulator of the human gut microbiota composition [15, 17–20] and that both total dietary fat and fat quality may shape the gut microbiota [21]. The complex interplay between diet and the gut microbiota leads to the growth of specialist microbes that produce metabolites, such as short-chain fatty acids (SCFAs), influencing host metabolism and cholesterol regulation [17, 22–24]. The type of fat seems to elicit distinct effects on gut microbiota. While high-fat diets containing SFAs have been shown to decrease bacterial diversity [25], unsaturated fat is reported to increase the total bacterial count [26]. A correlation between omega-3 (n3) fatty acids and gut microbiota [27, 28], including a positive correlation with bacteria of the *Lachnospiraceae* family [28, 29], has been reported. In a recent study in mice, an omega-6 (n6) high-fat diet induced changes in gut microbiota, including a reduction in *Lachnospiraceae* [30]. However, only a few studies have investigated the effect of fat quality on gut microbiota and the impact on human metabolic regulation.

Bile acids are produced from cholesterol in the liver and facilitate digestion and absorption of lipids. Bile acids may also have hormonal or signaling functions by binding to several receptors and thereby play a crucial role in metabolic regulation [31]. It is well known that gut microbiota can oxidize and degrade bile acids. Even though gut microbiota may affect metabolic regulation by conversion of bile acids and the interaction between gut microbiota and bile acid metabolism is an emerging topic, there are few clinical studies investigating the interaction between gut microbiota, bile acid metabolism, and risk of CVD [32].

In a recent publication, it was reported that exchanging intake of SFAs with PUFAs effectively reduced total cholesterol levels after only three days in healthy individuals [1]. Whether this effect could be related to changes in the gut microbiota is not known.

The aim of the present study was to investigate the effect of dietary fat quality on gut microbiota, SCFAs, and bile acids in healthy individuals and to explore the relationship between gut bacteria and blood lipids, bile acids, and fatty acids.

## Methods

### Subjects and study design

**Fig. 1:**
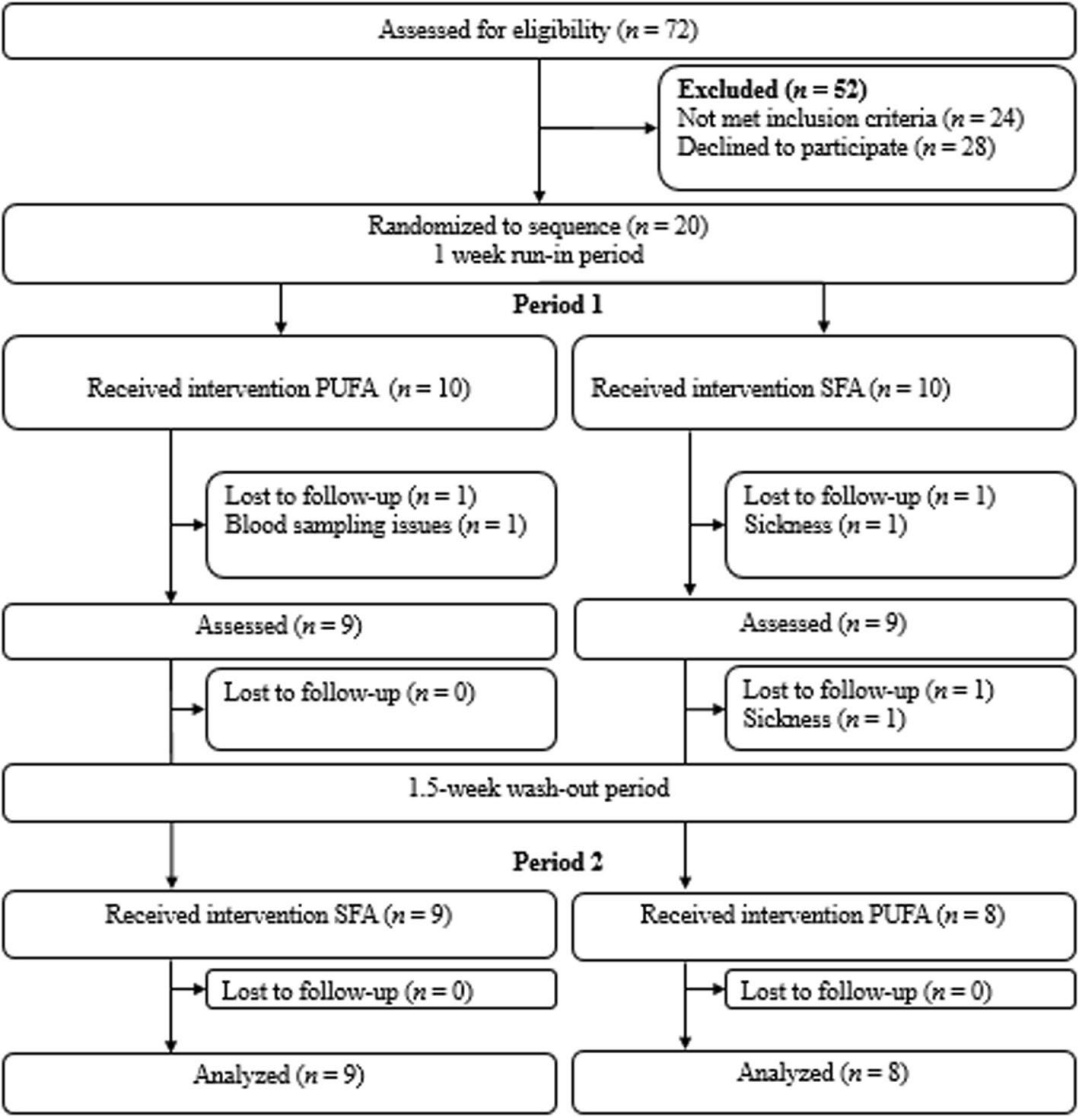
Flow chart of the participants included in the dietary crossover study (*n* = 17) [1]. Abbreviations: n: number; PUFAs: polyunsaturated fatty acids; SFAs: saturated fatty acids

The results from the present study were obtained from the same participants and set of samples as previously described [1]. Healthy volunteers (aged 18–65 years) with body mass index (BMI) between 18.5–27 kg/m^2^ were recruited from advertisements on Facebook, Oslo Metropolitan University (OsloMet) website, and from the student mass and employees at OsloMet to participate in a randomized controlled crossover study as described previously [1]. The study was performed between April 2018 and January 2019 at OsloMet. Inclusion and exclusion criteria have been described previously [1]. Briefly, the exclusion criteria were fasting blood glucose values ≥ 6.1 mmol/L, any food allergies or intolerances or chronic metabolic diseases (e.g., diabetes, CVD, and cancer), including the use of medication, intestinal diseases, including inflammatory bowel disease, celiac disease, and irritable bowel disease. Furthermore, those with high sensitivity C-reactive protein (hsCRP) > 10 mg/L, treated with antibiotics the previous three months and during the study, donating blood the previous two months or during the study, were pregnant or lactating, planned weight reduction and/or had ≥ 5% weight change the previous three months, used any hormonal treatment (except from oral contraception), tobacco, or had a high alcohol consumption (> 40 g/day) were excluded. Seventy-two volunteers were screened for participation, and 20 participants were willing to participate in the dietary crossover study, while 17 completed the study. Participants lost during follow-up and those included in the study are outlined in the flow chart (Fig. 1).

**Fig. 2:**
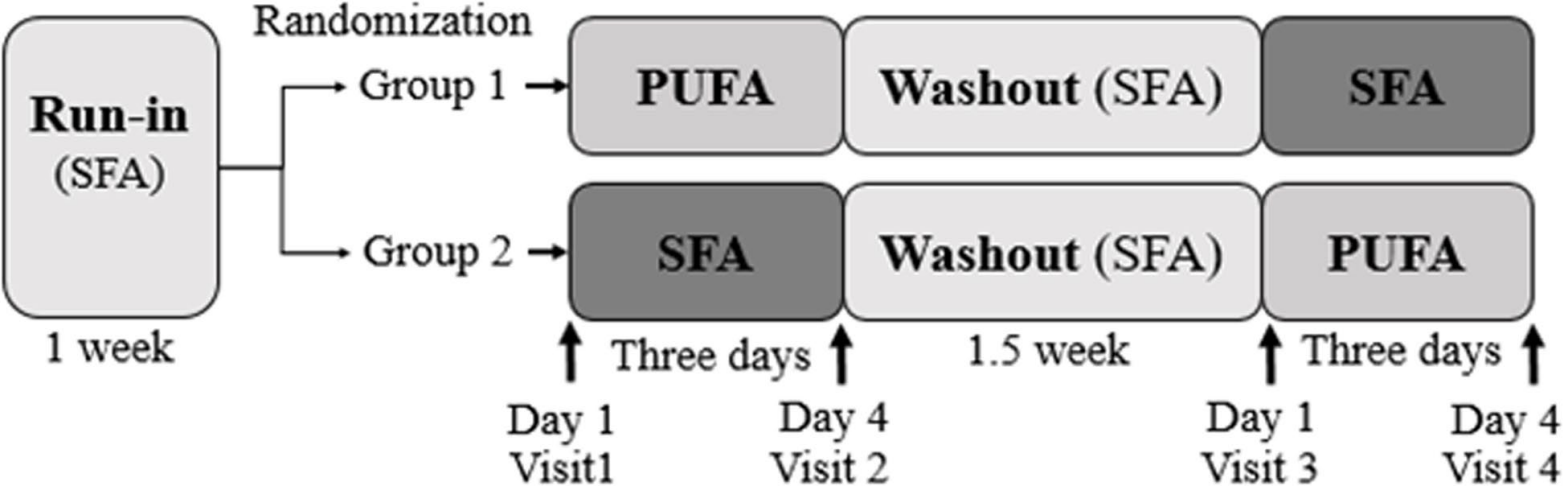
Study design of the double-blind, randomized, controlled crossover study. Described previously [1]. Seventeen healthy volunteers (group 1: *n* = 9; group 2: *n* = 8) received daily PUFA products (two muffins and 20 g margarine spread) or SFA products (two muffins and 20 g butter-based spread) for three consecutive days, separated by a 1.5-week washout period. The participants received SFA products in the run-in and washout periods. Fasted blood was measured for glucose, insulin, triglyceride, NEFAs, fatty acid profiles, SCFAs, bile acids, and total cholesterol at each visit. In addition, fecal samples were collected for gut microbiota analyses at each visit. Abbreviations: NEFAs: nonesterified fatty acids, PUFAs: polyunsaturated fatty acids, SCFAs: short-chain fatty acids, SFAs: saturated fatty acids

After a one-week run-in period, the participants were randomized (1:1 allocation ratio) and received either SFA products (muffins with butter-based spread and butter-based spread) (control) or PUFA products (muffins with margarine and margarine) for three consecutive days before crossing over to the other intervention, separated by a 1.5-week washout period (Fig. 2). In the run-in and washout periods, the participants received SFA (control) products. The study products (muffins and margarine or butter-based spread) replaced fat-containing products in their habitual diet. The fatty acid composition of the study products is given in Gaundal et al. [1]. The SFA products contributed 1085.5 kcal, 78.5 g carbohydrate, 13.4 g protein, 79.5 g total fat, 29.9 g SFAs, 33.4 g monounsaturated fatty acids (MUFAs) and 10.2 g PUFAs per day. The PUFA products contributed 985.8 kcal, 74.4 g carbohydrate, 13.9 g protein, 69.8 g total fat, 2.4 g SFAs, 30.9 g MUFAs and 26.4 g PUFAs per day [1].

The participants were advised to maintain their habitual diet and physical activity level throughout the study period, except for the dietary restrictions: limit their intake of dietary fats and products rich in beta-glucan, not taking dietary supplements (fish oil, probiotics, etc.) and antibiotics. Details about the study design, description of participants, and study products are described elsewhere [1].

All participants signed a written informed consent form prior to participation. The study was approved by the Regional Committees for Medical and Health Research Ethics (2018/104) and conducted according to the Declaration of Helsinki guidelines. The crossover study was registered at Clinical Trials (http://clinicaltrials.gov/, registration identification number: NCT03658681).

### Blood sampling and laboratory analyses

Blood samples were collected after an overnight fast (≥ 12 h) at each visit in the crossover study [1]. Fasting triglyceride and total cholesterol were measured in serum by a routine laboratory (Fürst Medical Laboratory) within 24 h. Furthermore, nonesterified fatty acids (NEFAs), fatty acid profiles and the SCFAs acetate, propionate and butyrate were measured in EDTA plasma in a commercial laboratory (Vitas Analytical Service, Oslo, Norway). NEFAs were measured using an enzymatic colorimetric assay with acyl-CoA oxidase and MEHA as color reagents. The total plasma (EDTA) fatty acid profile was measured with GC flame ionization detection. An internal standard (triheptadecanoin) was added, and the samples were methylated with 3 N HCl in methanol. Fatty acid methyl esters were extracted with hexane, and then samples were neutralized with 3 N KOH in water. After mixing and centrifuging, the hexane phase was injected into the GC flame ionizing detection. Analysis was performed on a 7890A GC with a split/splitless injector, a 7683B automatic liquid sampler and flame ionization detection (Agilent Technologies). Separations were performed on an SP-2380 (30 m × 0·25 mm i.d. × 0·25 μm film thickness) column from Supelco. The concentration of the individual fatty acids was measured as μg fatty acid/ml plasma and presented as a percentage of total fatty acids.

Nine bile acids, cholic acid (CA), deoxycholic acid (DCA), chenodeoxycholic acid (CDCA), taurocholic acid (TCA), taurodeoxycholic acid (TDCA), taurochenodeoxycholic acid (TCDCA), glycocholic acid (GCA), glycodeoxycholic acid (GDCA), and glycochenodeoxycholic acid (GCDCA), were measured in plasma using LC‒MS/MS with a sample volume of 50 µL and a single deuterium-labeled isotope as an internal standard. The plasma was initially treated with aqueous formic acid [1%] prior to protein precipitation with acetonitrile. The extracts were loaded onto a Hybrid SPE Phospholipid 96-well plate, and the effluent was dried in a vacuum centrifuge concentrator. LC‒MS/MS was performed on the reconstituted residues using a Shimadzu LC-20ADXR LC system coupled to a Sciex QTRAP5500 mass spectrometer with a Turbo V ion source and a TurboIonspray probe. Separation of the analytes was achieved using a Phenomenex Kinetex 2.6 μm Biphenyl 100A 100 × 4.6 mm column with an aqueous solution of ammonium acetate [5 mM] and acetic acid [2.1 mM] and an acetonitrile gradient mobile phase. Negative mode multiple reaction monitoring was used for detection. Linear calibration curves of the peak area ratios of the analyte and internal standard in aqueous methanol [50%] were used for quantification.

### Fecal collection and gut microbiota analyses

The participants received a sample collection kit (GA-map™ Dysbiosis Test, Genetic Analysis AS) for fecal collection and were instructed to sample the stool from three different places and place it in the sampling tubes. The samples were kept at room temperature for a maximum of three days before they were stored at -80 °C at OsloMet. The participants were instructed to sample the stool before (day 1) and after (day 4) each intervention, as close to the visits as possible. Fecal samples were collected at each visit. All samples were collectively sent to Genetic Analysis AS (GA) for microbiota analyses after the study was completed. The GA-map™ Dysbiosis Test is a commercially available genome-based test using fecal samples for analyses of gut bacteria, described in detail elsewhere [33]. The test comprises 48 DNA probes targeting ≥ 300 bacteria on different taxonomic levels, thus allowing mapping of the intestinal microbiota profile for a selected set of bacteria. To characterize and identify bacteria present, probes targeting seven variable regions (V3—V9) of the 16S rRNA gene were used. Human fecal sample homogenization and mechanical and enzymatic bacterial cell disruption were utilized to isolate and bind total bacterial genomic DNA to magnetic beads. The hypervariable regions V3—V9 of the 16S rRNA were further amplified by polymerase chain reaction (PCR). To determine bacterial DNA labeling, single nucleotide extension and hybridization to a complementary DNA strand were coupled to beads. The abundance of bacteria was assessed by the strength of the fluorescent signal (probe intensity) and measured by a Luminex 200 (Luminex Corporation). Of the 48 bacteria, 40 were detected in more than 50% of the participants at each timepoint and included in the analyses.

### Statistical analyses

The primary aim of the crossover study was to investigate the effect of fat quality on glycemic regulation, and the sample size was based on literature on fat and glycemic regulation as described previously [1], whereas secondary outcomes were fasting blood lipids, fatty acids, SCFAs, bile acids, and gut microbiota. Hence, the nature of the present study is explorative, and therefore correction for multiple testing has not been performed.

For the gut microbiota analyses, the relative abundance number was logarithmically transformed with base 2 (Log2) to reduce the skewness of the original data. The log2 ratio was obtained by calculating the ratio before and after intervention with SFAs or PUFAs. A paired t test was used to assess differences between the two interventions based on the log2 ratio. In addition, a paired t test was also used to calculate differences within each group (SFAs and PUFAs) based on the log2 values before and after intake of SFAs and PUFAs.

For the variables of SCFAs and bile acids, a paired sample comparison (Wilcoxon signed rank test) was used to assess differences between the two interventions (SFA and PUFA) and differences within each group. SCFA levels were reported as both absolute values and as a percent of the total SCFA level.

Associations between the change in blood parameters (lipids, fatty acids, bile acids) and the change in gut microbiota were assessed with Pearson correlation. *P* < 0.05 was regarded as statistically significant for all statistical analyses. Statistical analyses were performed with IBM SPSS statistics (version 27), and figures were designed with GraphPad Prism 8 for Windows (version 8.0.0.).

## Results

The participants were healthy and normal weight, with fasting glucose, hemoglobin A1c (HbA1c), triglyceride, total cholesterol, and diastolic and systolic blood pressure within the normal range (Table 1). Their baseline characteristics have previously been shown [1].

**Table 1:** Baseline characteristics of the participants, as previously shown [1]

		Median	25^th^—75^th^ percentiles
Male/female (*n*)	6/11		
Age (years)		28.0	25.0—46.0
BMI (kg/m^2^)		22.8	22.0—25.0
HbA1c (%)^a^		5.2	5.0—5.4
HbA1c (mmol/mol)^a^		33.0	31.2—35.5
Glucose (mmol/L)		5.1	4.9—5.4
Insulin (pmol/L)		51.0	31.0—60.0
Triglyceride (mmol/L)		0.9	0.6—1.4
Total cholesterol (mmol/L)		4.9	4.4—5.4
hsCRP (mg/L)		0.9	0.3—1.4
Systolic blood pressure (mmHg)^a^		123	113—136
Diastolic blood pressure (mmHg)^a^		71	66—75
Body fat percent (%)		24.4	12.5—33.9
FFM (kg)		47.8	45.3—69.3

^a^HbA1c, systolic and diastolic blood pressure measured at screening. Variables are measured fasted

*FFM* Fat free mass, *HbA1c* Hemoglobin A1c, *hsCRP* High sensitivity C-reactive protein

Whether intake of different fat qualities could affect the gut microbiota was further addressed. Of the 40 gut bacteria analysed, the abundance of *Bifidobacterium* spp. and *Lachnospiraceae* was significantly increased after intake of PUFAs compared to SFAs (*P* = 0.029 and *P* = 0.013, respectively) (Table 2). Furthermore, there was a tendency toward differences in the abundance of Actinobacteria, *Dialister invisus*, *Dialister invisus* & *Megasphaera micronuciformis, Eubacterium hallii* and *Phascolactobacterium* sp. between the interventions (Table 2).

**Table 2:** Differences in bacterial abundance after a three-day intervention with SFA and PUFA

	**SFA**			**PUFA**			
**Gut bacteria**	Day 1	Day 4	p^1^	Day 1	Day 4	p^1^	p^2^
*Actinobacteria* (A)	7.9 (1.1)	7.4 (1.1)	*0.087*	7.5 (1.2)	7.7 (1.3)	ns	*0.063*
*Actinomycetales *(A)	4.0 (1.1)	4.0 (1.0)	ns	4.4 (1.3)	4.2 (1.0)	ns	ns
*Bifidobacterium* spp. (A)	7.1 (1.3)	6.6 (1.3)	*0.091*	6.6 (1.3)	7.0 (1.4)	ns	**0.029**
*Alistipes* (B)	7.1 (1.8)	7.3 (1.8)	ns	7.2 (1.8)	7.5 (1.8)	**0.006**	ns
*Alistipes onderdonkii *(B)	4.2 (1.9)	4.3 (1.9)	ns	4.2 (1.9)	4.5 (2.1)	ns	ns
*Bacteroides fragilis *(B)	4.0 (2.0)	4.2 (2.1)	ns	3.8 (1.8)	4.1 (2.2)	ns	ns
*Bacteroides pectinophilus* (B)	3.6 (0.6)	3.5 (0.5)	ns	3.7 (0.7)	3.6 (0.7)	ns	ns
*Bacteroides* spp. (B)	5.3 (1.6)	5.4 (1.6)	ns	5.3 (1.5)	5.4 (1.5)	ns	ns
*Bacteroides* spp. & *Prevotella* spp. (B)	9.9 (0.4)	10.0 (0.3)	ns	9.9 (0.4)	9.9 (0.3)	ns	ns
*Bacteroides stercoris* (B)	3.8 (1.7)	3.9 (1.5)	ns	3.8 (1.5)	3.8 (1.4)	ns	ns
*Bacteroides zoogleoformans* (B)	3.7 (1.5)	4.1 (1.6)	*0.086*	3.9 (1.5)	3.9 (1.4)	ns	ns
*Parabacteroides johnsonii *(B)	3.9 (1.8)	4.0 (1.6)	ns	3.9 (1.8)	4.3 (1.8)	**0.014**	ns
*Parabacteroides* spp. (B)	5.5 (1.5)	5.6 (1.5)	ns	5.5 (1.5)	5.8 (1.6)	ns	ns
*Bacilli* (F)	7.2 (0.4)	7.1 (0.7)	ns	7.1 (0.8)	7.1 (0.5)	ns	ns
*Clostridia* (F)	8.9 (0.2)	9.0 (0.3)	ns	8.9 (0.3)	9.0 (0.2)	ns	ns
*Clostridium methylpentosum* (F)	4.1 (0.8)	4.2 (0.8)	ns	4.1 (0.8)	4.4 (0.9)	*0.082*	ns
*Coprobacillus cateniformis* (F)	2.9 (0.9)	2.8 (0.5)	ns	3.0 (1.0)	2.9 (1.2)	ns	ns
*Dialister invisus *(F)	7.1 (2.3)	7.3 (2.4)	ns	7.5 (2.3)	7.3 (2.2)	ns	*0.073*
*Dialister invisus *& *Megasphaera micronuciformis* (F)	5.6 (2.6)	5.8 (2.6)	**0.005**	5.9 (2.8)	5.8 (2.6)	ns	*0.067*
*Dorea* spp. (F)	4.5 (1.0)	4.5 (0.9)	ns	4.5 (1.2)	4.7 (0.9)	ns	ns
*Eubacterium hallii* (F)	6.6 (0.8)	6.3 (0.9)	*0.064*	6.3 (0.7)	6.5 (0.9)	ns	*0.095*
*Eubacterium biforme *(F)	5.0 (2.4)	4.9 (2.2)	ns	4.8 (2.2)	4.7 (2.2)	ns	ns
*Eubacterium rectale *(F)	6.2 (2.2)	6.2 (2.1)	ns	6.2 (2.0)	6.4 (2.0)	ns	ns
*Eubacterium siraeum *(F)	4.4 (1.1)	4.1 (1.1)	**0.043**	4.7 (0.8)	4.7 (1.2)	ns	ns
*Firmicutes* (F)	9.4 (0.4)	9.4 (0.6)	ns	9.5 (0.5)	9.4 (0.5)	ns	ns
*Faecalibacterium prausnitzii *(F)	8.8 (0.3)	8.7 (0.5)	ns	8.7 (0.3)	8.9 (0.3)	ns	ns
*Lachnospiraceae* (F)	9.8 (0.2)	9.8 (0.2)	ns	9.7 (0.3)	9.8 (0.2)	**0.009**	**0.013**
*Lactobacillus* spp. (F)	5.7 (2.3)	5.7 (2.0)	ns	5.5 (2.1)	5.5 (2.0)	ns	ns
*Lactobacillus *spp. 2 (F)	3.5 (1.0)	3.5 (1.2)	ns	3.2 (1.1)	3.4 (1.3)	ns	ns
*Phascolarctobacterium* sp. (F)	4.2 (1.9)	4.2 (1.8)	ns	4.1 (1.8)	4.2 (1.9)	*0.082*	*0.069*
*Ruminococcus albus* & *Ruminococcus bromii* (F)	4.9 (1.9)	5.0 (2.0)	ns	5.1 (1.9)	5.4 (2.0)	ns	ns
*Ruminococcus gnavus* (F)	4.0 (1.2)	4.0 (1.3)	ns	3.9 (1.1)	4.0 (1.1)	ns	ns
*Streptococcus agalactiae* & *Eubacterium rectale *(F)	5.9 (1.7)	5.9 (1.6)	ns	5.7 (1.7)	5.9 (1.5)	*0.065*	ns
*Streptococcus salivarius* *ssp. thermophilus* & *S. sanguinis *(F)	2.8 (0.4)	2.8 (0.5)	ns	3.4 (1.6)	3.3 (1.1)	ns	ns
*Streptococcus salivarius ssp. thermophilus *(F)	4.6 (1.4)	4.6 (1.4)	ns	4.7 (1.6)	4.8 (1.5)	ns	ns
*Streptococcus* spp. 2 (F)	3.5 (0.9)	3.4 (1.0)	ns	3.3 (0.8)	3.3 (0.8)	ns	ns
*Veillonella* spp. (F)	9.2 (0.4)	9.2 (0.4)	ns	9.2 (0.5)	9.2 (0.4)	ns	ns
Firmicutes various (F/T/B)	7.7 (1.5)	7.5 (1.4)	ns	7.4 (1.5)	7.6 (1.5)	ns	ns
*Proteobacteria* (P)	5.9 (0.3)	6.0 (0.8)	ns	6.0 (0.5)	6.0 (0.5)	ns	ns
*Mycoplasma hominis* (T)	6.3 (2.3)	6.3 (2.2)	ns	6.1 (2.2)	5.9 (2.3)	ns	ns

The values are log2 transformed and given as mean (SD). *P*-values indicate differences within (p^1^) or between (p^2^) SFA and PUFA interventions calculated with t-test

*A* Actinobacteria, *B* Bacteroidetes, *F* Firmicutes, *ns* Non-significant, *P* Proteobacteria, *PUFA* Polyunsaturated fatty acids, *SFA* Saturated fatty acids, *T* Tenericutes

**Fig. 3:**
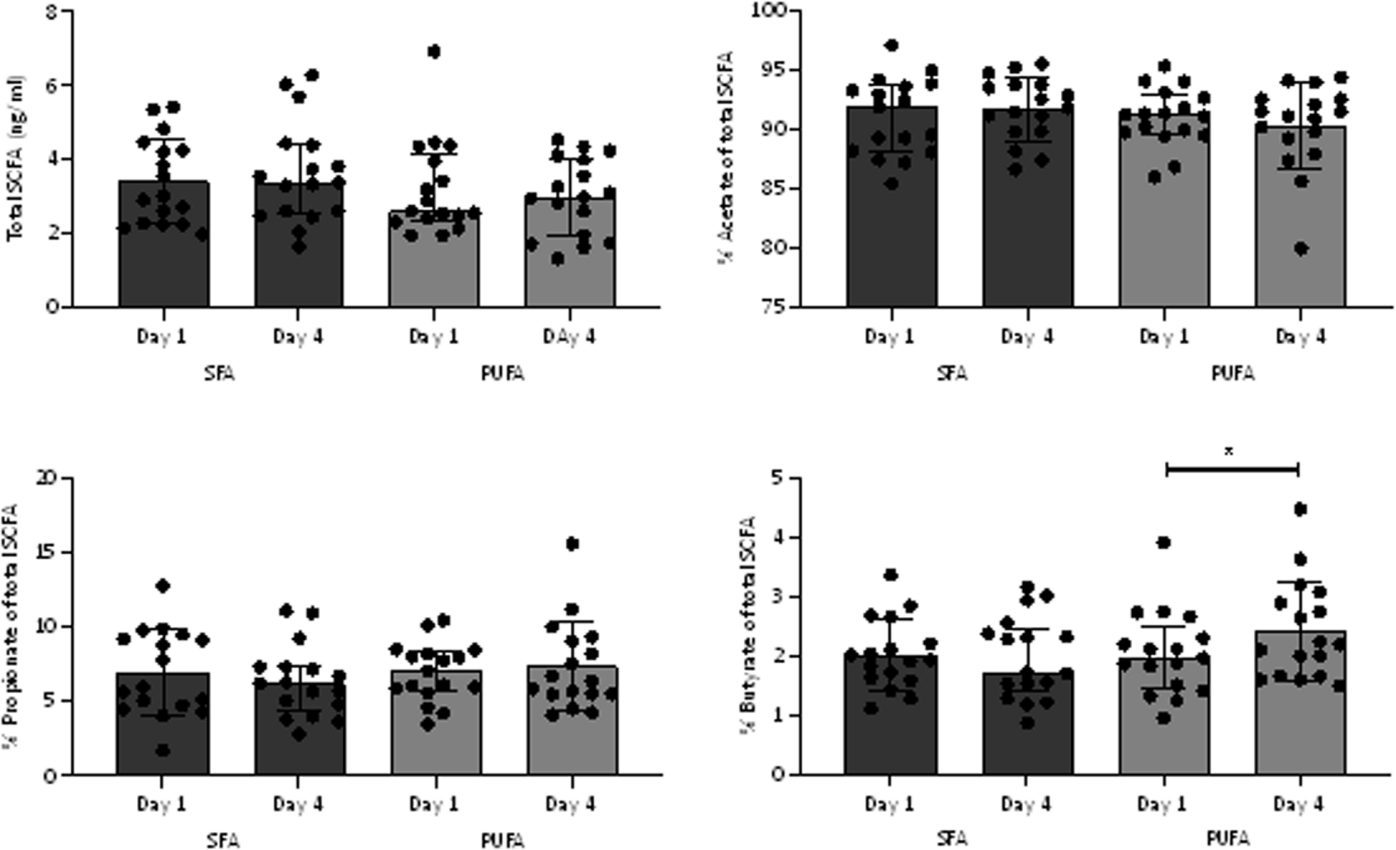
SCFAs before and after intake of SFAs or PUFAs. Total SCFAs and relative levels of acetate, propionate, and butyrate before (day 1) and after (day 4) intake of SFAs and PUFAs in healthy individuals (*n* = 17) for three consecutive days. Data are shown as the median, and bars indicate the 25th—75th percentiles. Within- and between-group differences were analyzed with the Wilcoxon signed rank test. * indicates *P* ≤ 0.05. Abbreviations: PUFAs: polyunsaturated fatty acids, SCFAs: short-chain fatty acids, SFAs: saturated fatty acids

The microbial metabolites SCFAs acetate, propionate and butyrate were measured in blood before and after intake of SFAs and PUFAs. There were no significant differences in total SCFA levels or relative levels of acetate, propionate, or butyrate between the interventions (Fig. 3). However, intake of PUFAs for three consecutive days significantly increased the relative level of butyrate (*P* = 0.015) (Fig. 3).

We have previously shown that total cholesterol levels significantly decreased after intervention with PUFAs compared with SFAs, whereas no significant effect on triglycerides and NEFAs was found [1]. As bile acids are synthesized from cholesterol, we wanted to investigate the effect of dietary fat on circulating levels of bile acids. Of the nine bile acids measured in plasma, no significant changes between the interventions were detected. Within interventions, however, changes were evident. Within the SFA intervention, TCA, GCA, and GCDCA decreased significantly (*P* = 0.039, *P* = 0.013, *P* = 0.028, respectively), while GDCA decreased and GCDCA increased within the PUFA intervention (*P* = 0.017, *P* = 0.025, respectively) (Table 3).

**Table 3:** Differences in bile acids after a three-day intervention with SFA and PUFA

	**SFA**			**PUFA**			
Bile acid (nM)	Day 1	Day 4	p^1^	Day 1	Day 4	p^1^	p^2^
Cholic acid (CA)	27.6 (20.2–111.2)	26.2 (12.2–299.0)	0.723	84.5 (14.2–239.4)	24.0 (8.7–83.5)	0.193	0.102
Deoxycholic acid (DCA)	244.9 (117.5–496.6)	275.0 (143.2–404.8)	0.332	253.0 (170.2–539.0)	207.7 (161.8–311.6)	0.113	0.124
Chenodeoxycholic acid (CDCA)	116.0 (61.9–194.9)	65.0 (35.9–284.6)	0.653	135.9 (65.3–315.7)	82.7 (17.0–216.4)	0.193	*0.068*
Taurocholic acid (TCA)	3.6 (1.5–7.8)	2.3 (0.6–4.1)	**0.039**	3.4 (1.9–14.5)	2.7 (1.4–8.3)	0.237	0.868
Taurodeoxycholic acid (TDCA)	26.9 (11.2–46.6)	18.9 (6.5–38.7)	0.177	25.6 (11.4–72.0)	17.7 (14.0–33.7)	0.193	0.586
Taurochenodeoxycholic acid (TCDCA)	46.6 (24.6–99.7)	43.1 (18.1–64.9)	*0.062*	60.3 (34.2–101.3)	54.6 (19.5–90.2)	0.163	0.831
Glycocholic acid (GCA)	47.4 (33.2–99.0)	32.3 (17.2–72.1)	**0.013**	58.3 (27.6–112.3)	53.8 (15.7–90.1)	0.287	0.869
Glycodeoxycholic acid (GDCA)	153.4 (76.4–236.8)	99.0 (50.5–205.8)	0.124	162.0 (74.2–285.3)	121.6 (62.9–165.5)	**0.017**	0.210
Glycochenodeoxycholic acid (GCDCA)	549.2 (219.6–724.7)	300.6 (222.3–491.1)	**0.028**	442.4 (248.6–763.0)	443.8 (108.8–585.9)	**0.025**	0.653

Data is given as median (25^th^—75^th^ percentiles). *P*-values indicate differences within (p^1^) or between (p^2^) SFA and PUFA interventions calculated with Wilcoxon signed rank test

*nM* Nanomolar, *PUFA* Polyunsaturated fatty acids, *SFA* Saturated fatty acids

**Fig. 4:**
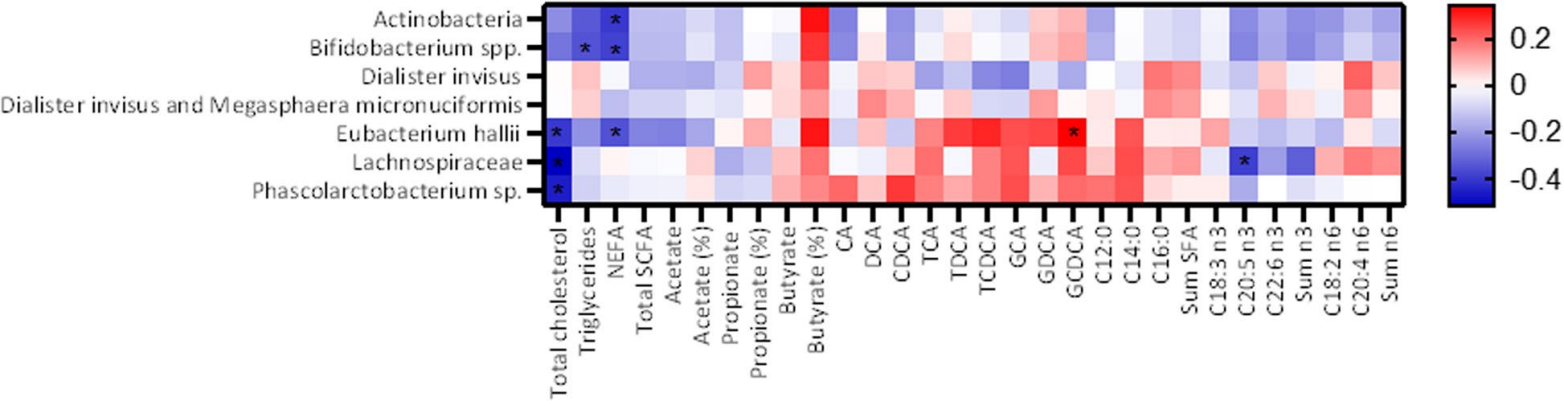
Relationship between changes in gut bacteria and total cholesterol, triglycerides, NEFAs, SCFAs, bile acids, and fatty acid profiles after intervention with SFAs and PUFAs. Correlation analysis was performed with Pearson correlation. * indicates *P* ≤ 0.05. Abbreviations: C12:0: lauric acid, C14:0: myristic acid, C16:0: palmitic acid, C18:3 n3: alpha-linolenic acid, C20:5 n3: eicosapentaenoic acid (EPA), C22:6 n3: docosahexaenoic acid (DHA), C18:2 n6: linoleic acid, C20:4 n6: arachidonic acid, CA: cholic acid, CDCA: chenodeoxycholic acid, DCA: deoxycholic acid, GCA: glycocholic acid, GDCA: glycodeoxycholic acid, GCDCA: glycochenodeoxycholic acid, n3: omega-3, n6: omega-6, NEFAs: nonesterified fatty acids, PUFAs: polyunsaturated fatty acids, SCFAs: short-chain fatty acids, SFAs: short-chain fatty acids, TCA: taurocholic acid, TDCA: taurodeoxycholic acid, TCDCA: taurochenodeoxycholic acid

**Fig. 5:**
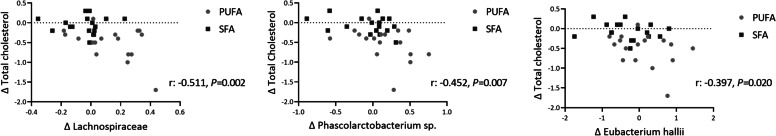
Relationship between the change in abundance of bacteria and total cholesterol level after interventions with SFAs and PUFAs. Correlation analysis was performed with Pearson correlation, and * indicates *P* ≤ 0.05. Abbreviations: PUFAs: polyunsaturated fatty acids, SFAs: saturated fatty acids

The gut bacteria that significantly differed or tended to differ after intervention with SFAs compared to PUFAs were thereafter correlated with the other variables. Changes in gut microbiota were associated with changes in total cholesterol levels, triglycerides, bile acids, and fatty acids (Fig. 4). We found a significant, negative correlation between the change in total cholesterol and the change in abundance of *Lachnospiraceae* (r: -0.511, *P* = 0.002), *Phascolarctobacterium* sp. (r: -0.452, *P* = 0.007), and *Eubacterium hallii* (r: -0.397, *P* = 0.020) (Figs. 4 and 5). There were also significant negative correlations between changes in NEFAs and changes in the abundance of Actinobacteria (r: -0.397, *P* = 0.020), *Eubacterium hallii* (r: -0.348, *P* = 0.044), and *Bifidobacterium* spp. (r: -0.379, *P* = 0.027) and between changes in triglycerides and *Bifidobacterium* spp. (r: -0.346, *P* = 0.045) (Fig. 4). Interestingly, we found that three bacteria (*Eubacterium hallii, Lachnospiraceae,* and *Phascolarctobacterium* sp.) correlated negatively with total cholesterol and positively with several of the bile acids. However, a significant positive correlation was only found between changes in the bile acid GCDCA and *Eubacterium hallii* (r: 0.343, *P* = 0.047) (Fig. 4). We also investigated correlations between the gut bacteria and fatty acids in blood. The change in *Lachnospiraceae* after intervention was negatively correlated with the change in eicosapentaenoic acid (EPA) (C20:5 n3) (r: -0.384, *P* = 0.025), but no other significant correlations were observed between the gut bacteria and fatty acids in blood (Fig. 4). Correlations between gender and age and the different variables were investigated. Age correlated significantly with *Eubacterium hallii* (r: 0.353, *P* = 0.040), *Eubacterium biforme* (r: 0.364, *P* = 0.034), *Eubacterium rectale* (r: -0.361, *P* = 0.036) and 20:4 n6 (r: 0.349, *P* = 0.043), while sex correlated significantly with *Bacilli* (r: -0.348, *P* = 0.043), *Actinomycetales* (r: -0.390, *P* = 0.022), *Shigella* spp. & *Echerichia* spp. (r: -0.346, *P* = 0.045), butyrate (r: 0.357, *P* = 0.038), and the bile acids GCA (r: -0.409, *P* = 0.016) and GCDCA (r: -0.408, *P* = 0.017).

## Discussion

In this study, the effect of different fat qualities (SFAs vs. PUFAs) on the human gut microbiota, SCFAs, and bile acids in healthy individuals was examined, in addition to the relationship between changes in gut bacteria and blood total cholesterol, triglycerides, NEFAs, bile acids, and fatty acid profiles. In a recent study, Gaundal et al. showed that replacing the intake of SFAs with PUFAs reduced total cholesterol levels by 8% after only three days in healthy individuals [1]. Accompanied by the reduced total cholesterol level, the present study demonstrates a shift in specific gut bacteria after replacing intake of SFAs with PUFAs for three days and identified specific bacteria associated with cholesterol levels in healthy individuals.

The abundance of *Lachnospiraceae* and *Bifidobacterium* spp. increased after three days following intake of PUFAs compared to SFAs, and the change in *Lachnospiraceae* was inversely associated with the change in total cholesterol levels. This is in line with a recent study showing that intake of PUFAs increased the abundance of *Lachnospira* and *Bifidobacterium* species in healthy individuals [34]. Findings from an animal study also showed similar results, where *Bifidobacterium* was negatively correlated with total cholesterol levels in hamsters [35]. Furthermore, oral administration of *Bifidobacterium* strains has been shown to elicit beneficial health effects, including lowering of total cholesterol levels both in mice [36] and humans [37, 38] and increasing the abundance of *Lachnospiraceae* [37]. Tindall and colleagues recently showed that members of the *Lachnospiraceae* family were inversely related to CVD risk factors, including total cholesterol, following a 6-week diet containing walnuts rich in PUFAs [39]. Consistent with these findings, the abundance of *Lachnospiraceae* was inversely associated with LDL cholesterol in patients with hyperlipidemia treated with rosuvastatin, a third-generation statin drug [40]. After dividing the patients into two groups based on their efficacy for rosuvastatin, those with high efficacy displayed a higher relative abundance of *Lachnospiraceae* compared to those with lower efficacy [40]. Taken together, the present results and findings from intervention trials might indicate that *Lachnospiraceae* and *Bifidobacterium* may act as cholesterol reducing agents.

**Fig. 6:**
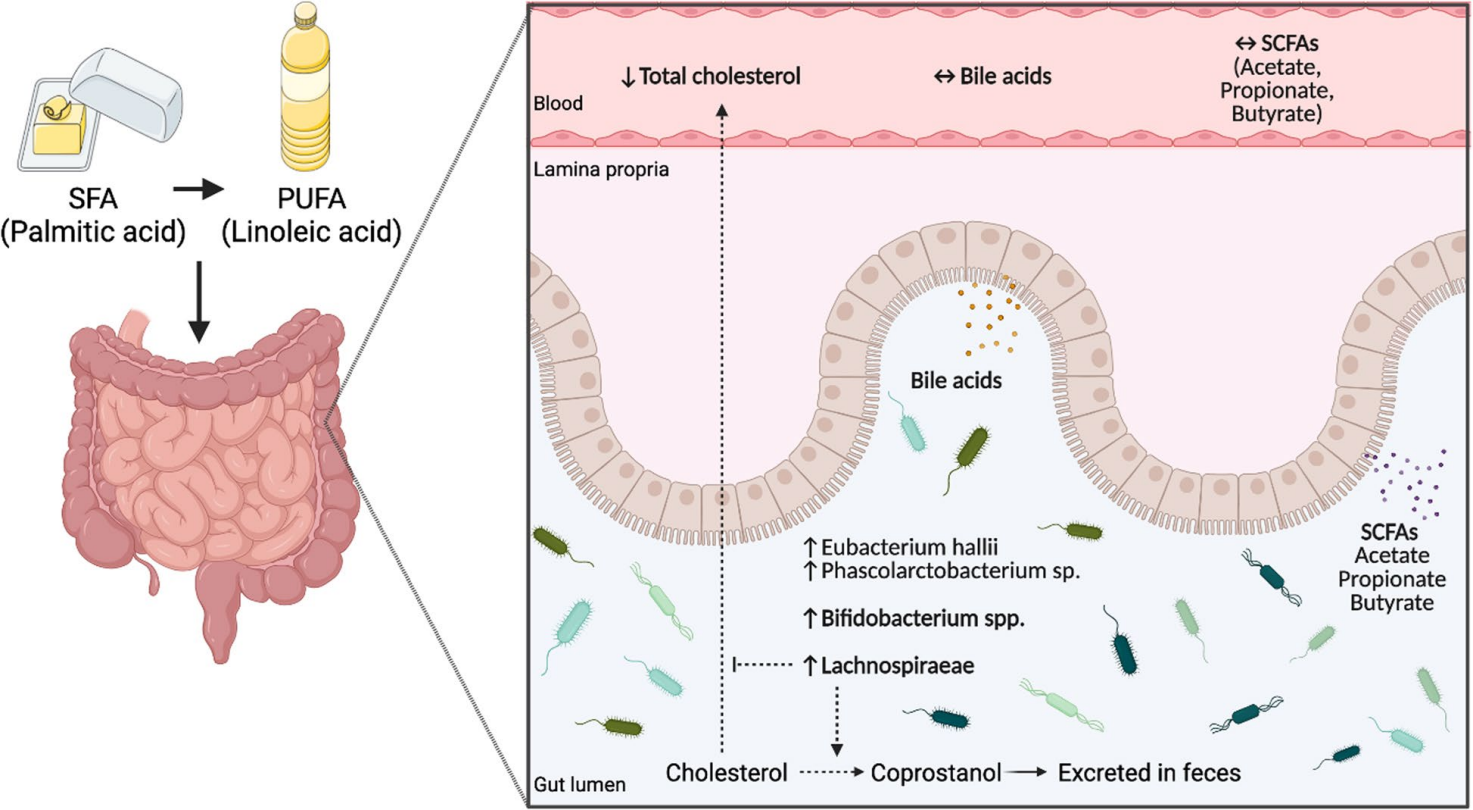
A graphic illustration of the hypothetical mechanisms related to fat quality, gut microbiota, and circulating cholesterol as outlined in the discussion. Replacing SFAs with PUFAs for three days did not affect the levels of bile acids and SCFAs in the circulation. However, gut bacteria may convert cholesterol in the intestine to coprostanol, which is secreted in feces, and hence the circulating levels of cholesterol will decrease. The figure was created with BioRender.com. Abbreviations: PUFAs: polyunsaturated fatty acids, SCFAs: short-chain fatty acids, SFAs: saturated fatty acids

Bile acids are formed from cholesterol in the liver, and increased synthesis and excretion of bile acids may affect cholesterol levels. This is the mechanism for the cholesterol reducing effect of bile acid binding resins. Even though a positive correlation between three bacteria and several bile acids was observed in this study, there was no significant effect on bile acids between interventions; hence, the cholesterol-lowering effect previously observed [1] may not be explained by changes in bile acids. Another explanation for the reduced cholesterol levels might be related to the bacterial conversion of cholesterol to coprostanol, a nonabsorbable sterol [41] (Fig. 6). Approximately 1 g of cholesterol originating from diet and bile enters the colon each day and interferes with colonic bacteria that reduce cholesterol to coprostanol [23]. Coprostanol is poorly absorbed by human intestinal cells and is subsequently excreted via feces [42]. Even though intestinal cholesterol conversion was discovered more than a century ago [41], only limited knowledge about cholesterol metabolism by the gut microbiota exists. In an early study, Wilson found that rats fed palmitic acid (C16:0) or oleic acid (C18:0) had decreased coprostanol formation, while rats fed n6 linoleic acid (C18:2n6) had increased formation of coprostanol from cholesterol [43]. This is in line with the present study, where the exchange of fatty acids was mainly from the SFA palmitic acid to linoleic acid [1]. Linoleic acid is well recognized to decrease cholesterol levels [2, 44, 45], but whether this effect might partially be caused by gut microbiota is still to be elucidated. Other studies also suggest that gut microbes play an important role in regulating serum cholesterol levels. In one study, they showed an inverse relationship between serum cholesterol levels and coprostanol and cholesterol levels in feces [42], and in a recent study by Kenny et al., they identified microbial cholesterol dehydrogenase enzymes and found an association between cholesterol-metabolizing bacteria and reduced serum cholesterol levels [46]. In the present study, an inverse relationship between *Lachnospiraceae*, *Eubacterium hallii* and *Phascolarctobacterium* sp. and total cholesterol levels are demonstrated after replacing intake of SFAs with PUFAs for three days. Interestingly, conversion of cholesterol to coprostanol has previously been shown after only three days [47]; however, the rate of this conversion in humans seems to be highly variable [23, 48]. In line with the present results, most of the cholesterol-reducing bacteria involve members of *Eubacterium*, but strains of *Bifidobacterium* and bacteria belonging to the *Lachnospiraceae* family are also reported to reduce cholesterol to coprostanol [23, 49, 50]. These findings suggest that specific gut bacteria may influence cholesterol metabolism via conversion of cholesterol to coprostanol, but whether this is the case in this study is not known. Therefore, more studies are needed to further elucidate the gut bacterial mechanisms involved in cholesterol regulation.

The current findings indicate that fat quality elicits different microbial changes, irrespective of the total fat amount. This is in line with a study by Caesar and colleagues, who showed that mice fed lard and fish oil had different effects on the gut microbiota composition and diversity [21]. Interestingly, the same group reported an interaction between dietary lipids and the gut microbiota in the regulation of hepatic cholesterol metabolism [51]. They showed that in mice fed lard, but not fish oil, the gut microbiota increased hepatic levels of cholesterol and cholesteryl esters. Furthermore, a recent systematic review of randomized controlled trials and observational studies showed that a high fat intake, and in particular a high intake of SFAs, had unfavorable effects on human gut microbiota richness and diversity [52]. In the present study, the fatty acid composition of the study products differed mainly in exchanging palmitic acid with linoleic acid, while n3 fatty acids did not differ between the interventions [1]. There were no correlations between gut bacteria and n6 fatty acids, but there was a negative correlation between EPA and *Lachnospiraceae*. Selmin et al. found a reduction in *Lachnospiraceae* after a n6 high-fat diet in mice [30]. Others have shown a positive correlation between circulating levels of docosahexaenoic acid (DHA) (C22:6n3) and *Lachnospiraceae* [29] and an increase in several butyrate-producing bacteria in the *Lachnospiraceae* family after intervention with a n3 fatty acid-rich diet in adult males [27]. The *Lachnospiraceae* family consists of several butyrate-producing bacteria [53]. Even though a significant increase in circulating relative butyrate levels within the PUFA intervention was observed in this study, there were no differences in total SCFAs or butyrate between the interventions or a correlation with *Lachnospiraceae.* SCFAs are well-known fermentation products from fiber and have been shown to have positive health effects [8]. However, the present study does not indicate that fat and fat quality affect SCFA fermentation.

Taken together, the effect of total fat intake and fat quality on gut microbiota and *Lachnospiraceae* is not clear. Nevertheless, fat quality may be a promising factor affecting host metabolism by altering the composition and function of the gut microbiota, but robust clinical studies are warranted to investigate this further.

## Study strengths and limitations

The randomized controlled crossover design strengthens the findings in the present work, as it corrects for any biological and interindividual differences between participants. This is important when investigating gut microbiota that are known to have large interindividual variation. However, there are some limitations to the study. First, due to the explorative nature of the study, we did not correct for multiple testing. Second, targeted analysis of the gut microbiota, including a panel of 48 bacteria, was performed (in which 40 bacteria were included in statistical analyses), and thus, from this study, it is not known whether other bacteria not included in this panel may impact cholesterol metabolism. The most common bacteria found in the human gut were measured, and specific bacteria associated with cholesterol levels were identified, in line with other studies. However, the *Lachnospiraceae* family consists of several bacterial species, and there is a need to further investigate which of the bacteria in this family may impact cholesterol levels. Third, even though diet has been shown to affect gut microbiota within one to three days, the short-term duration of the present study (three days per intervention) does not reflect the long-term effects. Fourth, the participants in the present study were normal weight, healthy adults (median age of 28 years); thus, the results cannot be generalized to the population as a whole.

## Conclusion and perspectives

In this study, the abundance of the gut bacterial families *Lachnospiraceae* and *Bifidobacterium* spp. was increased after replacing SFAs with PUFAs for three days in healthy individuals. Furthermore, the increase in *Lachnospiraceae* was associated with reduced total cholesterol levels. Whether the reduction in cholesterol levels is linked to linoleic acid-driven coprostanol formation by gut microbiota needs to be elucidated. Future robust studies are needed to investigate whether fat quality may be a promising factor affecting circulating cholesterol levels in the host by altering the composition and function of the gut microbiota. Dietary manipulation of the gut microbiota might be important for lipid-lowering prevention and treatment in the future.

## Data Availability

The datasets used and/or analyzed during the current study are available from the corresponding author on reasonable request.

## Abbreviations

A: Actinobacteria
B: Bacteroidetes
CA: Cholic acid
CDCA: Chenodeoxycholic acid
CVD: Cardiovascular diseases
DCA: Deoxycholic acid
DHA: Docosahexaenoic acid
EPA: Eicosapentaenoic acid
F: Firmicutes
FFM: Fat free mass
GCA: Glycocholic acid
GDCA: Glycodeoxycholic acid
GCDCA: Glycochenodeoxycholic acid
HbA1c: Hemoglobin A1c
hsCRP: High sensitivity C reactive protein
n: Number
n3: Omega 3
n6: Omega 6
NEFA: Nonesterified fatty acid
ns: Nonsignificant
P: Proteobacteria
PUFA: Polyunsaturated fatty acid
RCT: Randomized controlled trial
SCFA: Short-chain fatty acid
SFA: Saturated fatty acid
T: Tenericutes
TCA: Taurocholic acid
TDCA: Taurodeoxycholic acid
TCDCA: Taurochenodeoxycholic acid
